# Glucagon-like-peptide-1-Rezeptoragonisten: eine neue pharmakologische Behandlungsoption für psychiatrische Erkrankungen?

**DOI:** 10.1007/s00115-025-01813-x

**Published:** 2025-03-05

**Authors:** Hubertus Himmerich

**Affiliations:** 1 Centre for Research in Eating and Weight Disorders (CREW), Department of Psychological Medicine, Institute of Psychiatry, Psychology & Neuroscience, King’s College London, 16 De Crespigny Park, SE5 8AB London, Großbritannien; 2 South London and Maudsley NHS Foundation Trust, London, Großbritannien; 3 Klinik für Psychiatrie, Psychotherapie und Psychotraumatologie, Bundeswehrkrankenhaus Berlin, Berlin, Deutschland

**Keywords:** Diabetes, Demenz, Binge-eating-Störung, Depression, Abhängigkeitserkrankungen, Diabetes, Dementia, Binge eating disorder, Depression, Substance use disorders

## Abstract

Zu den Glucagon-like-peptide-1(GLP-1)-Rezeptoragonisten gehören Albiglutid, Dulaglutid, Exenatid, Liraglutid, Lixisenatid, Orforglipron und Semaglutid. Tirzepatid bindet nicht nur an GLP-1-, sondern auch an glukoseabhängige insulinotrope Peptidrezeptoren (GIP). Retratrutid ist ein dreifacher GLP-1-, GIP- und Glucagonrezeptoragonist. GLP-1-Rezeptoragonisten erhöhen die Insulin- und unterdrücken die Glucagonfreisetzung. Sie verlangsamen die Magenentleerung und verhindern so Blutzuckerspitzen. Sie reduzieren den Appetit und die Nahrungsaufnahme. Im Gehirn führen GLP-1-Rezeptoragonisten zu einer besseren Blutzuckerkontrolle, und sie scheinen entzündungshemmende und neuroprotektive Wirkungen zu haben. Es wurde berichtet, dass GLP-1-Rezeptoragonisten oxidativen Stress und apoptotische Prozesse reduzieren, das Risiko einer Ischämie senken und die Neurogenese fördern. GLP-1-Rezeptoragonisten können auch die dopaminerge Signaltransduktion im Nucleus accumbens beeinflussen. Somit könnten sie die Wirkung von Kokain, Alkohol und Nikotin verändern. Vorläufige Untersuchungen weisen auf einen therapeutischen Nutzen von GLP-1-Rezeptoragonisten für Patientinnen und Patienten mit Demenz, Essstörungen, psychopharmakologisch induzierter Gewichtszunahme, Depressionen, Angststörungen und Abhängigkeitserkrankungen hin. Typische unerwünschte Begleiterscheinungen sind gastrointestinale Nebenwirkungen wie Übelkeit, Erbrechen, Durchfall, Aufstoßen und gastroösophagealer Reflux. Zu den schwerwiegenderen Nebenwirkungen gehören Pankreatitis, allergische Reaktionen, Nierenfunktionsstörungen und möglicherweise ein erhöhtes Schilddrüsenkarzinomrisiko.

## Abstract (en)

Albiglutide, dulaglutide, exenatide, liraglutide, lixisenatide, orforglipron and semaglutide are glucagon-like peptide‑1 (GLP-1) receptor agonists. Tirzepatide targets not only GLP‑1 but also glucose-dependent insulinotropic peptide (GIP) receptors and retatrutide is a triple GLP‑1, GIP and glucagon receptor agonist. The GLP‑1 receptor agonists increase insulin release but suppress glucagon release. They slow down the emptying of the stomach and thus prevent blood sugar spikes. They reduce appetite and food intake. In the brain GLP‑1 receptor agonists lead to a better glycemic control and they appear to have anti-inflammatory and neuroprotective effects. It has been reported that GLP‑1 receptor agonists reduce oxidative stress and apoptosis, lower the risk of ischemia and promote neurogenesis. The GLP‑1 receptor agonists can also influence dopaminergic signal transduction in the nucleus accumbens. Therefore, they could modify the effect of cocaine, alcohol and nicotine. Preliminary investigations provide indications of the therapeutic benefits of GLP‑1 receptor agonists for people with dementia, eating disorders, psychopharmacologically induced weight gain, depression, anxiety and substance use disorders. Typical accompanying adverse reactions are gastrointestinal side effects, such as nausea, vomiting, diarrhea, eructation and gastroesophageal reflux. More severe side effects include pancreatitis, allergic reactions, renal function disorders and possibly an increased risk of thyroid cancer.

## GLP-1-Rezeptoragonisten zur Behandlung von Diabetes und Adipositas

Die Glucagon-like-peptide-1(GLP-1)-Rezeptoragonisten werden zur Behandlung von Typ-2-Diabetes (T2D) und Adipositas eingesetzt. Sie waren im Jahr 2024 die meistverkaufte Medikamentenklasse mit einem geschätzten weltweiten Jahresumsatz von ~50 Mrd. US-Dollar.

In den frühen 2000er-Jahren belegten randomisierte kontrollierte Studien (RCTs) die Wirksamkeit der GLP-1-Rezeptoragonisten Liraglutid und Semaglutid in der Behandlung von T2D [1]. Darüber hinaus zeigte sich, dass sie zu Gewichtsabnahme führen. Es folgten RCTs, die eine signifikante Gewichtsabnahme bei Patientinnen und Patienten mit Adipositas zeigten [2]. Während Liraglutid noch tägliche Injektionen erforderte, da es schnell durch Peptidasen metabolisiert wird, muss Semaglutid nur einmal wöchentlich injiziert werden. Mittlerweile ist Semaglutid sogar als orale Tablette erhältlich [3].

Anfang der 2020er-Jahre wurde Tirzepatid getestet, das nicht nur an GLP‑1, sondern auch an glukoseabhängigen insulinotropen Peptidrezeptoren (GIP) bindet. Während die herkömmlichen GLP-1-Rezeptoragonisten zu einem Gewichtsverlust von ca. 15 % führen, erreicht Tirzepatid bei einer wöchentlich injizierten Dosis von 10 mg oder 20 mg einen Gewichtsverlust von ~20 % [4]. Retratrutid, ein GLP-1-, GIP- und Glucagonrezeptoragonist, führt sogar zu durchschnittlich 24 % (~26 kg) Gewichtsverlust innerhalb von 48 Wochen, verglichen mit 2 % unter Placebo [5].

Die neueste Entwicklung ist Orforglipron, das nicht peptidbasiert ist und oral eingenommen wird [6]. Tab. 1 listet GLP-1-Rezeptoragonisten, ihre Wirkung auf verschiedene Rezeptoren, ihre Handelsnamen, ihre Indikation und ihren Zulassungsstatus, ihre Einnahmedosis und -häufigkeit auf.

**Tab. 1:** GLP-1-Rezeptoragonisten, Handelsnamen, Verabreichungsweg, Einnahmehäufigkeit und Dosis

GLP-1-Rezeptoragonist	Rezeptoragonismus	Handelsname	Indikation und Zulassung	Verabreichung	Häufigkeit	Dosis	Anmerkung
Albiglutid	GLP‑1	Tanzeum®, Eperzan®	T2D^⦸^	s.c.	Wöchentlich	30 oder 50 mg	2018 vom Markt genommen
Dulaglutid	GLP‑1	Trulicity®	T2D^☑^	s.c.	Wöchentlich	0,75 bis 4,5 mg	–
Exenatid	GLP‑1	Byetta®	T2D^☑^	s.c.	2 × täglich	5 bis 10 μg	Kurzwirksame Injektion von Exenatid
		Bydureon®	T2D^☑^	s.c.	Wöchentlich	2 mg	Langwirksame Depotinjektion von Exenatid
Liraglutid	GLP‑1	Victoza®	T2D^☑^	s.c.	Täglich	0,6 bis 1,8 mg	–
		Saxenda®	Adipositas^☑^	s.c.	Täglich	0,6 bis 3 mg	Nicht erstattungsfähig
Liraglutid plus Insulin degludec (fixe Kombination)	GLP-1, Insulin	Xultophy®	T2D^☑^	s.c.	Täglich	0,36 bis 1,8 mg Liraglutid; 10 bis 50 Einheiten Insulin degludec	2016 in Deutschland vom Markt genommen
Lixisenatid	GLP‑1	Adlyxin® Lyxumia®	T2D^☑^	s.c.	Täglich	10 bis 20 μg	2014 in Deutschland vom Markt genommen
Lixisenatid plus Insulin glargin (fixe Kombination)	GLP-1, Insulin	Suliqua®	T2D^☑^	s.c.	Täglich	5 bis 20 μg Lixisenatid; 10 bis 40 Einheiten Insulin glargin	Fixkombination von Insulin glargin und Lixisenatid
Orforglipron	GLP‑1	Noch kein Handelsname	Noch keine Indikation^⦸^	Oral	Täglich	12 bis 45 mg	Nicht auf Peptidbasis, nicht zugelassen
Retatrutid	GLP‑1, GIP, Glucagon	Noch kein Handelsname	Keine Zulassung^⦸^	s.c.	Wöchentlich	4 bis 12 mg	Nicht zugelassen
Semaglutid	GLP‑1	Ozempic®	T2D^☑^	s.c.	Wöchentlich	0,25 bis 2 mg	–
		Wegovy®	Adipositas^☑^	s.c.	Wöchentlich	0,25 bis 2,4 mg	Nicht erstattungsfähig
		Rybelsus®	T2D^☑^	Oral	Täglich	3 bis 14 mg	Nicht bei Adipositas zugelassen, nur bei T2D
Tirzepatid	GLP‑1, GIP	Mounjaro®	T2D^☑^	s.c.	Wöchentlich	2,5 bis 15 mg	–
			Adipositas^☑^	s.c.	Wöchentlich	2,5 bis 15 mg	Nicht erstattungsfähig
		Zepbound®	Adipositas^⦸^	s.c.	Wöchentlich	2,5 bis 15 mg	Tirzepatid ist bisher nur unter dem Handelsnamen Mounjaro® in Deutschland zugelassen

*GLP‑1* „glucagon-like-peptide-1“, *GIP* glukoseabhängiges insulinotropes Peptid, *®* Kennzeichnung als Handelsname, ^⦸^ keine Zulassung in Deutschland, ☑ in dieser Indikation in Deutschland zugelassen, *T2D* Typ-2-Diabetes, *s.c.* subkutane Injektion

GLP-1-Rezeptoragonisten vermitteln die Wirkung des Hormons GLP‑1, welches der Darm nach dem Essen natürlicherweise produziert. GLP-1-Rezeptoragonisten binden an GLP-1-Rezeptoren, die die Freisetzung von Insulin stimulieren. Sie unterdrücken außerdem die Glucagonsignalübertragung, verlangsamen die Magenentleerung und reduzieren Appetit und Nahrungsaufnahme.

Manche GLP-1-Rezeptoragonisten sind als fixe Kombination mit unterschiedlichen Insulinarten erhältlich. Beispielsweise ist die Kombination von Lixisenatid plus Insulin glargin unter dem Handelsnamen Suliqua® in Deutschland zugelassen und verfügbar. Xultophy®, die Kombination von Liraglutid mit Insulin degludec, ist in anderen europäischen Ländern verfügbar, wurde aber in Deutschland aus wirtschaftlichen Gründen vom Markt genommen.

## Unterschiede zwischen den GLP-1-Rezeptoragonisten

Daraus ergibt sich, dass sich GLP-1-Rezeptoragonisten nach ihrem Rezeptorprofil, ihrer Einnahmefrequenz und ihrem Einnahmeweg unterscheiden lassen.

- *Rezeptorprofil*: Albiglutid, Dulaglutid, Exenatid, Liraglutid, Lixisenatid, Orforglipron und Semaglutid sind reine GLP-1-Rezeptoragonisten. Tirzepatid ist ein GLP-1- und GIP-Rezeptoragonist, und Retatrutid ist ein GLP-1-, GIP- und Glucagonrezeptoragonist. Tirzepatid und Retatrutid führen entsprechend zu einer stärkeren Gewichtsabnahme als die reinen GLP-1-Rezeptoragonisten [5, 6].
- *Einnahmeweg*: Albiglutid, Dulaglutid, Exenatid, Liraglutid, Lixisenatid, Tirzepatid und Retratrutid werden subkutan injiziert. Semaglutid kann sowohl subkutan injiziert als auch oral eingenommen werden. Orforglipron wird oral eingenommen, ist allerdings noch nicht auf dem Markt erhältlich.
- *Einnahmefrequenz*: Exenatid als kurzwirksame Injektion, Liraglutid, Lixisenatid, Orforglipron und orales Semaglutid müssen täglich eingenommen werden, während Albiglutid, Dulaglutid, Exenatid als langwirksame Injektion, Tirzepatid und Retratrutid einmal wöchentlich injiziert werden.

## Reduktion der körperlichen Folgeerkrankungen

Zusätzlich zu den positiven Auswirkungen auf den Blutzuckerspiegel und das Körpergewicht bei Patientinnen und Patienten mit T2D und Adipositas wurde berichtet, dass GLP-1-Rezeptoragonisten das Risiko für Schlaganfall [7], Herzinfarkt und Tod durch kardiovaskuläre Ursachen senken [8]. Darüber hinaus könnten sie auch nützliche Medikamente gegen Dyslipidämie und nichtalkoholische Fettlebererkrankungen sein [9]. Es wurde gezeigt, dass GLP-1-Rezeptoragonisten bei T2D die Albuminurie reduzieren und die glomeruläre Filtrationsrate verbessern [10].

## GLP-1-Rezeptoragonisten und psychische Erkrankungen

GLP-1-Rezeptoren werden nicht nur im peripheren Körper, sondern auch im Gehirn exprimiert, beispielsweise im Frontallappen, im Hippokampus und im Hypothalamus [11]. GLP-1-Rezeptoragonisten beeinflussen insbesondere Zentren und Botenstoffe der Appetitregulation. Beispiele sind hypothalamische Neuropeptid- und Hormonsignale, glutamaterge Signale im Locus coeruleus (LC), cholecystokininexprimierende Neurone und das Melanocortinsystem im Hirnstamm [12].

Präklinische Studien konnten zeigen, dass sie durch verschiedene Mechanismen Nervenzellen schützen, z. B. durch die Hemmung von entzündlichen Prozessen, oxidativen Schäden und Apoptose [13]. Interessanterweise wurde in Tierstudien festgestellt, dass GLP-1-Rezeptoragonisten den Drang zum Konsum von Alkohol unterdrücken [14].

GLP-1-Rezeptoragonisten könnten besonders bei der Behandlung psychischer Störungen von Nutzen sein, die mit Adipositas assoziiert sind. Die Adipositas ist ein Risikofaktor für die Entwicklung einer Demenz, und die Adipositas ist oft die Folge einer Binge-eating-Störung (BES) oder das Resultat einer Gewichtszunahme unter psychopharmakologischer Behandlung, und sie tritt oft komorbid mit Depressionen und Angststörungen auf [12].

## Demenz

Es wurde berichtet, dass GLP-1-Rezeptoragonisten eine neuroprotektive Wirkung haben, indem sie Entzündungsprozesse hemmen, die durch hohe Glukosespiegel getriebene Neurodegeneration bremsen und die neuronale Insulinresistenz verbessern. Daher werden sie derzeit als mögliche Behandlung der Alzheimer-Krankheit, der Parkinson-Krankheit und der vaskulären Demenz diskutiert [11, 13]. Eine retrospektive Kohortenstudie mit Daten von 5 Mio. adipösen erwachsenen Patientinnen und Patienten, von denen über 100.000 mit GLP-1-Rezeptoragonisten behandelt wurden, errechnete Unterschiede im Auftreten einer neurodegenerativen Erkrankung zwischen adipösen Patientinnen und Patienten, die eine GLP-1-Rezeptoragonisten-Behandlung erhielten, und adipösen Patientinnen und Patienten, die keine GLP-1-Rezeptoragonisten-Behandlung bekamen. Es zeigte sich eine signifikante Risikoreduktion bei Patientinnen und Patienten unter GLP-1-Rezeptoragonisten bezüglich der Alzheimer-Erkrankung, der Lewy-Körper-Demenz und der vaskulären Demenz. Für die vaskuläre Demenz wurde das relative Risiko durch GLP-1-Rezeptoragonisten-Behandlung mehr als halbiert [15].

In diesen Indikationen fehlen jedoch publizierte RCTs. Es werden allerdings RCTs zu Liraglutid [16] und Semaglutid [17] bei Alzheimer-Erkrankung durchgeführt.

## Binge-eating-Störung

Eine offene placebokontrollierte Pilotstudie untersuchte die Wirkung von Liraglutid bei Patienteninnen und Patienten mit Adipositas und Essattacken. Teilnehmer, die Liraglutid erhielten, zeigten eine signifikante Verbesserung der Essattacken und eine Verringerung des Körpergewichts und der Glukoseplasmakonzentration [18]. Eine retrospektive Studie zu den Auswirkungen von Semaglutid auf die BES berichtete über ähnliche Ergebnisse in der Semaglutid-Gruppe im Vergleich zu Studienteilnehmern, die Lisdexamfetamin (LDX) oder Topiramat erhielten [19]. LDX und Topiramat sind die von der World Federation of Societies of Biological Psychiatry (WFSBP) empfohlenen pharmakologischen Behandlungsoptionen für BES, wenn psychotherapeutische Maßnahmen nicht greifen oder nicht vorhanden sind [20].

Bisher wurde nur eine RCT veröffentlicht, in der der GLP-1-Rezeptoragonist Liraglutid bei Patientinnen und Patienten mit BES getestet wurde. Obwohl in der BES-Gruppe ein statistisch signifikanter Gewichtsverlust festgestellt wurde, muss das Studienergebnis mit Vorsicht interpretiert werden, da ein Fehler bei der Medikamentenabgabe aufgetreten war und nur 27 Patientinnen und Patienten an dieser Studie teilnahmen [21].

## Gewichtszunahme während psychopharmakologischer Behandlung

Im Hinblick auf Gewichtszunahme und Adipositas während einer psychopharmakologischen Behandlung zeigte eine RCT mit 103 Teilnehmern, die mit Clozapin oder Olanzapin behandelt wurden und übergewichtig oder adipös waren, dass Liraglutid die Glukosetoleranz verbessert und das Körpergewicht reduziert [22]. Diese Ergebnisse werden durch offene Studien bestätigt, die einen signifikanten Gewichtsverlust unter Exenatid oder Liraglutid bei adipösen oder übergewichtigen Patientinnen und Patienten unter Behandlung mit Antipsychotika festgestellt haben [23].

## Depression und Angsterkrankungen

Depressionen und Adipositas treten häufig komorbid auf. Kalorienreduzierte Ernährung führt dagegen bei Patientinnen und Patienten mit Depression und Adipositas sowohl zu Gewichtsverlust als auch zu einer Verbesserung der depressiven Symptome [24]. Eine aktuelle Metaanalyse mit ~2000 Teilnehmern in 5 RCTs ergab, dass Erwachsene, die mit GLP-1-Rezeptoragonisten behandelt wurden, im Vergleich zu denen, die mit Kontrollsubstanzen behandelt wurden, eine signifikante geringere depressive Symptomatik aufwiesen [25]. Die genauen biologischen und psychologischen Mechanismen, die diesem Effekt zugrunde liegen, sind noch unklar.

Eine aktuelle Auswertung von Daten aus Ambulanzen und Krankenhäusern, die die Epic-Krankenhaussoftware nutzen, untersuchte einen möglichen Zusammenhang zwischen GLP-1-Rezeptoragonisten und psychischen Erkrankungen nach Beginn der Medikamenteneinnahme. Die Autoren werteten dazu Daten von ~3 Mio. Diabetikern und ~1 Mio. Nichtdiabetikern aus. Unter den Patientinnen und Patienten mit Diabetes zeigten diejenigen, denen eines der untersuchten GLP-1-Medikamente (mit Ausnahme von Liraglutid) verschrieben wurde, geringere Wahrscheinlichkeiten für das Auftreten einer Depression oder einer Angsterkrankung im Vergleich zu denen, denen kein GLP-1-Rezeptoragonist verschrieben wurde. Tirzepatid zeigte z. B. eine Verringerung der Auftretenshäufigkeit einer Depression bei Diabetikern um 65 % [26]. Einschränkend ist anzumerken, dass diese Datenauswertung bislang nicht in einer Zeitschrift mit Begutachtungsverfahren publiziert wurde.

## Abhängigkeitserkrankungen

In einer landesweiten Kohortenstudie in Dänemark mit Fallserienanalyse wurde untersucht, ob die Einnahme von GLP-1-Rezeptoragonisten mit dem Risiko alkoholbedingter Ereignisse verbunden ist. Dazu wurden ~40.000 T2D-Patientinnen und -Patienten, die GLP-1-Rezeptoragonisten einnahmen, mit ~50.000 Patientinnen und Patienten, die Dipeptidylpeptidase-4(DPP4)-Inhibitoren einnahmen, verglichen. Während der Nachbeobachtungszeit von durchschnittlich 4,1 Jahren war eine GLP-1-basierte Behandlung im Vergleich zur DPP4-Behandlung mit einem geringeren Risiko eines alkoholbedingten Ereignisses verbunden (Hazard Ratio: 0,46; [27]).

In einer RCT bei Patientinnen und Patienten mit Alkoholabhängigkeit veränderte der GLP-1-Rezeptoragonist Exenatid jedoch nicht den Alkoholkonsum [28].

## Methodenkritische Bewertung der Studien zu GLP-1-Rezeptoragonisten bei psychischen Erkrankungen

Die Evidenzlage zur Wirksamkeit von GLP-1-Rezeptoragonisten unterscheidet sich deutlich zwischen den verschiedenen psychiatrischen Indikationen und den unterschiedlichen GLP-1-Rezeptoragonisten. Metaanalytische Evidenz besteht für die Verbesserung einer depressiven Symptomatik unter Exenatid und Liraglutid bei Patientinnen und Patienten mit Adipositas und T2D [25]. RCT-basierte Evidenz besteht für die Gewichtsabnahme unter Liraglutid bei übergewichtigen oder adipösen Patientinnen und Patienten, die mit Clozapin oder Olanzapin behandelt wurden [22]. Allerdings handelt es sich lediglich um eine einzige RCT, sodass die abgeleitete Evidenz auch als limitiert angesehen werden muss. Weitere Details zur Evidenzlage bezüglich der Wirksamkeit von GLP-1-Rezeptoragonisten bei psychischen Erkrankungen sind in Tab. 2 dargestellt.

**Tab. 2:** Potenzielle psychiatrische Indikationen, getestete GLP-1-Rezeptoragonisten und höchste Evidenz geordnet nach Evidenzgrad

Indikation	GLP-1-Rezeptoragonisten	Höchste Evidenz	Anmerkungen und Limitationen
Depressive Symptomatik	Exenatid (+) Liraglutid (+)	Metaanalyse [25]	Metaanalyse auf Grundlage von 5 RCTs und einer prospektiven Kohortenstudie
			Die depressive Symptomatik war nicht die Hauptindikation
Antipsychotikainduzierte Gewichtszunahme	Liraglutid (+)	RCT [22]	Signifikant weniger schwerwiegende unerwünschte Nebenwirkungen in der Liraglutid-Gruppe vs. Placebo
			Keine Unterschiede in der Lebensqualität oder der Schwere der psychiatrischen Erkrankung
Binge-eating-Störung	Liraglutid (+)	RCT [21]	In dieser RCT-Studie unterlief ein Medikationsfehler
Alkoholabhängigkeit	Exenatid (−)	RCT [28]	Trotz des insgesamt negativen Ergebnisses ergaben explorative Analysen, dass Exenatid die Tage mit starkem Alkoholkonsum und den Gesamtalkoholkonsum in der Untergruppe adipöser Patientinnen und Patienten signifikant reduzierte
Demenz	Semaglutid (+) Dulaglutid (−) Liraglutid (−)	Kohortenstudie [15]	Positive Evidenz für die Gruppe der GLP-1-Rezeptoragonisten und speziell für Semaglutid
			Keine Evidenz für Dulaglutid und Liraglutid, die auch untersucht wurden
Prophylaxe von depressiven Störungen und Angsterkrankungen	Semaglutid (+) Tirzepatid (+)	Auswertung klinischer Datenbanken [26]	Diese Studie wurde bislang nicht in einer Zeitschrift mit Begutachtungsverfahren publiziert
			Die Relevanz der Effekte für Dulaglutid, Exenatid und Liraglutid ist unklar
			Die primären Behandlungsindikationen waren T2D und Adipositas, nichtdepressive Störungen und Angsterkrankungen

*RCT* randomisierte kontrollierte Studie, + positive Evidenz, − kein Unterschied zur Vergleichsgruppe oder negative Evidenz

## Nebenwirkungen

Die häufigsten Nebenwirkungen von GLP-1-Rezeptoragonisten sind gastrointestinale Beschwerden wie verminderter Appetit, Übelkeit, Erbrechen, Aufstoßen, Bauchschmerzen und Verstopfung. Sie treten vor allem bei Therapiebeginn und bei Dosissteigerungen auf. Pankreatitis und Gallenerkrankungen können ebenfalls auftreten [29]. Eine aktuelle Metaanalyse klinischer Studien zeigte einen moderaten Anstieg des relativen Risikos und einen geringen Anstieg des absoluten Risikos für Schilddrüsenkarzinome [30].

In den letzten Jahren wurde in der Presse immer wieder darüber berichtet, dass nichtadipöse Menschen, darunter auch Prominente, Semaglutid zur kosmetischen Gewichtsabnahme missbrauchen. Eine Analyse des FDA (United States Food and Drug Administration) Adverse Events Reporting System ergab, dass tatsächlich im Vergleich zu ähnlichen Medikamenten ein Missbrauchspotenzial bestehen könnte [31]. Des Weiteren wächst die Besorgnis, dass Patientinnen und Patienten mit restriktiven Essstörungen wie der Anorexie oder der atypischen Anorexie krankheitsbedingt GLP-1-Rezeptoragonisten anwenden könnten [32].

Aufgrund der Besorgnis über Suizidalität unter der Behandlung mit GLP-1-Rezeptoragonisten wurde vom FDA Adverse Event Reporting System eine Analyse anhand von Daten, die zwischen 2005 und Oktober 2023 erhoben worden waren, durchgeführt. Der Bericht ergab, dass nur Suizidgedanken häufiger gemeldet wurden, jedoch kein suizidales Verhalten, Suizidversuche oder vollendete Suizide [33]. Eine aktuelle Metaanalyse von 31 RCTs, in denen GLP-1-Rezeptoragonisten getestet wurden, konnte weder erhöhte psychische Gesundheitsprobleme noch ein erhöhtes Suizidrisiko feststellen [34].

Wichtige pharmakologische Effekte, aktuell aufgrund präklinischer und klinischer Daten diskutierte mögliche Indikationen und Nebenwirkungen der GLP-1-Rezeptoragonisten zeigt Tab. 3.

**Tab. 3:** Synopsis der pharmakologischen Effekte, der aktuell aufgrund präklinischer und klinischer Daten diskutierten möglichen Indikationen und Nebenwirkungen von GLP-1-Rezeptoragonisten^a^

Periphere pharmakologische Effekte	Zentralnervöse pharmakologische Effekte	Potenzielle oder schon zugelassene internistische Indikationen	Potenzielle neurologische Indikationen	Potenzielle psychiatrische Indikationen	Nebenwirkungen
Insulin↑ Glucagon↓ Magenentleerung↓	Insulinresistenz↓ Oxidativer Stress↓ Tau-Hyperphosphorylation↓ Β‑Amyloidablagerung↓ Neurogenese↑ Neuroinflammation↓ Neuroprotektion↑ Mitochondrale Dysfunktion↓ Apoptose↓	Adipositas Koronare Herzerkrankung Dyslipidämie Nichtalkoholische Fettleber Typ-2-Diabetes	Amyotrophe Lateralsklerose Autoimmunenzephalitis Chronisches Schmerzsyndrom Idiopathische intrakranielle Hypertonie Ischämischer Schlaganfall Morbus Parkinson Multiple Sklerose Periphere Neuropathie Traumatische Hirnverletzung	Abhängigkeitserkrankungen Alzheimer- und vaskuläre Demenz Binge-eating-Störung Angststörungen Depressive Störungen Psychopharmakologisch induzierte Gewichtszunahme	Aufstoßen Bauchschmerzen Durchfall Erbrechen Gallengangserkrankungen Nierenprobleme Missbrauch bei Anorexie oder Bulimie Pankreatitis Übelkeit Schilddrüsenkarzinome^b^ Verminderter Appetit Verstopfung Zystitis

^a^Für erklärende Informationen und Literaturbelege siehe Text und [11, 12, 26]

^b^Das Risiko für Schilddrüsenkarzinome ist unklar

## Fazit für die Praxis

- Die zugelassenen Glucagon-like-peptide-1(GLP-1)-Rezeptoragonisten sind hochwirksame Substanzen gegen Typ-2-Diabetes (T2D) und Adipositas.
- Ihre Verschreibung allein zur Adipositasbehandlung ist laut Gemeinsamem Bundesausschuss (G-BA) nicht erstattungsfähig.
- GLP-1-Rezeptoragonisten haben möglicherweise ein therapeutisches Potenzial für Demenz, Binge-eating-Störung (BES), depressive Störungen, Angststörungen, Suchterkrankungen und eine psychopharmakologisch induzierte Gewichtszunahme.
- Metaanalysen legen keine erheblichen psychiatrischen Nebenwirkungen nahe.
- Kritisch sind ein möglicherweise leicht erhöhtes Risiko für Schilddrüsenkarzinome und die nicht medizinisch indizierte Einnahme von GLP-1-Rezeptoragonisten zu kosmetischen Zwecken zu sehen.
